# Understanding Attitudes, Social Norms, and Behaviors of a Cohort of Post-Operative Nurses Related to Pain and Pain Management

**DOI:** 10.3390/healthcare10050844

**Published:** 2022-05-04

**Authors:** Nur Pinar Ayaz, Deborah Witt Sherman

**Affiliations:** 1 Nursing Department, Faculty of Health Sciences, Hitit University, Corum 19200, Turkey; nayaz001@fiu.edu; 2 Nicole Wertheim College of Nursing and Health Sciences, Florida International University, Miami, FL 33199, USA

**Keywords:** nurses, pain assessment, pain management, culture, attitudes, norms, behaviors, opioids

## Abstract

Despite advances in surgical techniques and peri-operative care, pain is a significant symptom post-operatively. The purpose of this study was to examine the attitudes, social norms, and behaviors of a cohort of nurses of various ethnic and cultural backgrounds regarding pain and pain assessment and management. The design was a descriptive qualitative study guided by Theory of Planned Behavior and the Theory of Transcultural Nursing. The sample included six registered nurses (RNs) (two Hispanic, two African American, and two Caucasian), along with patients of the same and different cultural and ethnic backgrounds in the post-operative unit within 48 h of surgery. The nurses’ results indicated that nurses assess pain severity and patient treatment preferences yet do not conduct a comprehensive pain assessment and have limited knowledge of non-pharmacologic and complementary pain therapies. Despite knowledge of the patient’s pain history, tolerance, and cultural background, the nurses believed that “all patients should be treated the same” and were concerned about the use of opioids beyond the first 24–48 h post-operatively due to the risk of addiction and professional ramifications related to opioid administration. The conclusions are that ongoing education is needed regarding comprehensive pain assessment and pharmacologic, non-pharmacologic, and complementary pain therapies during the post-operative period. Discussion is needed regarding the importance of diversity and equity as it relates to cultural competence within the context of pain assessment and management to provide patient-centered individualized care.

## 1. Introduction

Pain, which is one of the oldest and most complex health problems, is an unpleasant sensory and personal experience with physical, emotional, social, and cultural characteristics that adversely affect the patient’s functioning and quality of life [1,2]. Despite technological advances in pharmacology, surgical techniques, and peri-operative care, pain is a significant symptom experienced by surgical patients [3], with 86% reporting the pain as severe [4]. Post-operative pain is experienced at the highest level within the first 36 hours following surgery and gradually decreases by the third postoperative day [3,5,6].

As pain management is a fundamental right of the patient [7], patients expect that health professionals, especially nurses, will actively monitor and relieve pain with pharmacologic, non-pharmacologic, and complementary therapies. Nurses’ attitudes regarding pain, the assessment of pain, and decisions regarding pain management are important, given the realization that inadequate pain assessment results in inadequate pain management [8,9]. When post-operative pain is not well controlled, it may cause various physiological and psychological effects, as well as increase the risk of acute pain turning into chronic pain [10]. Inadequate post-operative pain control also increases the cost of care by delaying hospital discharge and prolonging the healing process [4].

Since pain is a subjective experience, if a person says that he or she has pain, it should be accepted as true [11]. Yet, it is known that nurses’ and patients’ attitudes toward pain and pain management are affected by the person’s past pain experience, age, and familial and cultural reactions to pain [8,12]. One of the challenges in providing healthcare in the US is caring for patients of diverse cultural backgrounds and ethnic groups, who may have similar or different attitudes and perceptions related to pain [13,14,15]. Although patients may have the same surgical procedure and the same pain stimulus, they also may have very different pain tolerance levels and, given their cultural backgrounds, may express pain differently [8,15]. While nurses and patients of the same cultural background may have similar attitudes, differences in cultural background may increase the likelihood of patients’ pain not being properly evaluated and treated [8,15]. There are no studies in the literature that focus on the similarities and differences of attitudes, social norms, and behaviors of nurses and patients of the same or different cultural or ethnic backgrounds. The purpose of this study was therefore to explore the similarities and differences of nurse and post-operative patient dyads of the same or differing cultural and ethnic backgrounds regarding attitudes, social norms, and behaviors related to pain and pain management. This article focuses on the results of interviews of the cohort of nurses in the post-operative observation unit.

### 1.1. Research Question

In qualitative research, a research question states the phenomenon of interest and the population of interest. In this study, the research question was posed not as a declarative statement but as an interrogative question, specifically “What are the attitudes, social norms, and behaviors of culturally diverse nurses related to pain and pain management who are caring for patients during the post-operative period?”.

### 1.2. Conceptual Framework

This study was guided by the Theory of Planned Behaviors [16], which is a socio-psychological approach purporting that an individual’s intention toward behavior can be estimated by his or her attitudes and social norms, which may include cultural expectations regarding a behavior [17]. The Theory of Transcultural Nursing by Leininger (1999) further emphasizes the importance of cultural values, beliefs, practices, and attitudes in the care of patients and the effect of nurses’ awareness of their own cultural influences on pain assessment and management [18,19].

## 2. Methods

### 2.1. Research Design

Given that there were no instruments with published reliability and validity to measure the concepts of this study, it was decided that the study design would be a descriptive qualitative study. This design may be guided by a conceptual framework which informs the development of structured interview questions. In this study, the interview guide asked about attitudes, social norms, and behaviors related to pain and pain management. As a qualitative study, the Standards for Reporting Qualitative Research (SRQR) informed the research process and critical appraisal criteria [20].

### 2.2. Researcher Characteristics and Reflexivity

The principal investigator (PI), as an international doctoral student, was funded by the Republic of Turkey Ministry of National Education. The Republic of Turkey Ministry of National Education stipulated that the study was to involve surgical patients, with the recommendation to study some aspect of pain and pain management. Both the PI and second author had surgical nursing experience, which informed the development of the research question and the study proposal. Their clinical expertise on post-operative units also informed the development of the interview questions and interpretation of the data.

### 2.3. Sample and Setting

The nursing manager of the post-operative unit introduced the PI to the nursing staff to discuss the study. A flyer with the contact name of the researcher was distributed and posted to the unit to promote study recruitment. The purposive sample consisted of a cohort of 6 registered nurses (RNs) (2 Hispanic, 2 African American, and 2 Caucasian) who cared for patients within 48 h of surgery in the post-operative observation unit of a large tertiary medical system. Although the inclusion of Asian nurses was sought, there were no Asian nurses employed in the post-operative unit. The nurses’ inclusion criteria were the following: registered nurses (RNs) of identified ethnic or cultural backgrounds who worked in a post-operative unit, spoke English, and worked 12-h day shifts. The exclusion criteria included RNs who did not provide direct post-operative care. The ethnic groups identified in this study aligned with the ethnic groups identified by the National Institutes of Health in the US. Ethnicity refers to groups of people depending on their ancestry, social and cultural identity, race, language, country of birth, or other special characteristics of the society in which they were born, while culture is a social phenomenon that is generally based on the morals, values, beliefs, and characteristics of a particular group [8]. As a purposive sample, the sample was intentionally selected to represent nurses of differing ethnic or cutural backgrounds. In terms of the setting, unlike post-anesthesia care (PACU), where there can be a 1:1 nurse-to-patient ratio due to the acuity of the patients, who are often admitted to the hospital and transferred to a surgical intensive care unit, the post-operative observation unit of this facility was selected, as patients were being recovered who were not expected to be admitted to the hospital but, due to co-morbid conditions or potential operative complications, may stay in the unit for up to 48 h post-operatively for observation.

### 2.4. Human Subject Protection and Data Collection Procedures

This study was conducted in accordance with the Declaration of Helsinki. Study approval was obtained from the Institutional Review Board (IRB) of Florida International University (FIU IRB-20 0137, approved on 12 July 2020) and the IRB of Baptist Hospital (BH IRB 20 0137-AM01, approved on 10 November 2020).

Informed consent was obtained from all subjects involved in the study. Upon signing the informed consent, the participants completed a Demographic and Professional Data Form. Digitally recorded interviews were conducted in a private office in the unit, lasting approximately 45 min. To ensure confidentiality, the participants were assigned a code number to all study materials. The study data were kept in the PI’s locked file cabinet and a password-protected computer.

### 2.5. Data Collection Instruments

A structured interview guide was developed based on the conceptual framework of the study and was informed by the surgical experiences of the authors. The interview guide was reviewed and revised based on the feedback of six master’s-prepared nursing students enrolled in a research project course taught by the second author.

### 2.6. Data Processing, Units of Study, and Analysis

The sample of six nurses completed the Demographic and Professional Data Form, which was analyzed using descriptive statistics, and six interviews were digitally recorded and sent electronically via secure measures to a transcription company for transcription. The transcripts, as well as the researcher logs and analytic memos, were analyzed and coded according to Carini’s principles [21], specifically (1) developing detailed knowledge of the interview content, (2) reviewing the researcher’s logs and analytic memos, (3) listing tentative headings and reflecting on recurring ideas, (4) analyzing verbatim statements and listing them under identified headings with the grouping of similar concepts, (5) summarizing new impressions, (6) comparing commonalities and differences in the participants’ statements, and (7) establishing themes that describe the patterns and observations of the interview of the individual participants at the data collection point on case-by-case analysis and across the interviews of the participants. Trustworthiness was established by developing an audit trail and through peer debriefing. The research team coded the transcripts independently and then discussed the coding and emerging themes to establish inter-rater reliability. Member checking occurred as the study results were shared with two participants who verified the results.

## 3. Results

The nurses’ ages ranged between 20 and 39 years, with 5 out of 6 reporting being female and single. Three of the nurses were born in the US, while the others were from Haiti, Cuba, and Guyana. They were of varying religious backgrounds. All of the nurses had a bachelor’s degree, worked as an RN for between 1 to 7 years, and worked from 2 months to 5 years in the post-operative observation unit. All the nurses stated that they did not receive formal training in pain assessment and management in their BSN programs. However, post-degree, half completed continuing education courses on pain assessment and management and received ongoing training in culture competence. In reporting the findings, pseudonyms were given to the nurse participants rather than the use of their initials.

### 3.1. Attitudes Related to Pain Assessment and Management

#### 3.1.1. Do Nurses Expect Post-Op Pain?

All the nurses stated that they expect pain in the post-operative period and that the pain would gradually decrease. However, they emphasized the importance of assessing pain intensity and cited their responsibility to act on the patient’s behalf by giving pain medications, advocating for acceptable pain control, and educating patients about what to expect following surgery. Nurse Clara said *“Post-operative pain is expected. I explain that they are not going to be completely absent from pain, but I will help reduce it.”*

#### 3.1.2. What Does Pain Tell You or Mean?

The nurses emphasized that pain is a subjective feeling which indicates that something is physically or psychologically wrong and necessitates a prioritization of patients in pain. Each believed that if a patient says he or she has pain, he or she should not be judged, and individualized care should be given. The nurses knew that patients reacted differently to pain due to differences in pain thresholds and tolerance levels. The nurses recognized that some people express pain verbally or through non-verbal gestures, such as facial grimacing. Nurse Alexandra said that *“It’s very subjective. Some people have more tolerance for pain, while others are less tolerant. People experience pain differently, and you should not judge anyone.”*

#### 3.1.3. What Pain Reactions Do Nurses Consider Inappropriate?

The nurses emphasized that they are concerned if a patient screams, yells, curses, or is demanding and aggressive, as it is “rude” to the healthcare team and shows “inappropriate behavior”. Nurse Clara stated that *“It’s when patients are yelling, screaming, cursing, that kind of behavior needs to be immediately addressed.”* The nurses said that patients who report frequent pain are trying to attract attention, but they do not judge them. The nurses believed that if a patient asks frequently for pain medication, they need to learn about other pain medications or ways of reducing their anxiety. Nurse Amanda said that *“I try not to judge them, like if they’re in pain and they require more pain medication, but it is important to follow the orders and hospital rules.”* Nurse Clara emphasized that *‘’I just try to educate them, but it’s not our job to say you’re asking for it too much, if they have it ordered.”*

#### 3.1.4. How Important Are Nurses’ Interactions with Patients Post-Operatively?

All of the nurses recognized that developing a caring relationship with a patient is important in providing post-operative care, but that time is limited when patients only remain in the observation unit for 48 h. During this time, the nurses emphasized that supportive interactions included “trying to calm the patient by giving them attention and even changing the environment by decreasing noise and increasing privacy.” In return, the nurses felt that they “gained the patient’s cooperation” and that the patients were “more likely to follow the nurse’s instruction or advice.” The nurses spoke of the “demands that were made by some patients who were not understanding of their workloads and work stress” and that some patients were “constantly on the call bell”, while others “saw how busy the nurses were with other patients who were in severe pain”. Nurse Clara was concerned that *“When patients pressure nurses to move quickly, we are at greater risk of making mistakes.”*

#### 3.1.5. Does a Patient’s Substance Abuse History Affect Post-Operative Care?

The nurses were concerned that “patients may exaggerate their pain because they have a substance abuse history and are drug seeking”. Another nurse realized that “if a patient has had an opioid addiction, they may need more pain medications because they have a higher level of tolerance”. The majority of the nurses took a “neutral” and “nonjudgmental” approach, realizing patients also have “different personalities” and nurses are “responsible for minimizing pain”.

#### 3.1.6. What Are Nurses’ Perceptions Regarding the Impact of Pain on Patients’ Physical Abilities, Psychological Well-Being, or Family Interactions?

All the nurses recognized that pain restricts patient functions and movements, including activities of daily living, and with limited mobility due to pain, patients are at “greater risk for post-op complications, such as pneumonia, DVT, muscle atrophy, and alterations in the overall nervous system. Such restrictions and problems can also delay the healing process and recovery.” On a psychological level, the nurses cited that “patients in pain may be agitated, anxious, withdrawn, and at times, even ‘crazy’.” Pain affects “patients’ ideals and moral function” and may lead to a “decreased mood, increased stress, depression, hopelessness, as well as less willingness to cooperate”. The nurses also recognized the various relationships between pain and interactions with family. Depending on the patient and family, their communication may differ. “Some families worry too much about the patient and this may increase the anxiety of the patient.” Nurse Alexandra stated that *“I notice some patients are better without the family interaction, as they follow instructions better when family is not present.”*

#### 3.1.7. Does a Patient’s Verbal Pain Reporting Affect the Nurses’ Post-Operative Pain Management?

All of the nurses agreed that the patients’ verbal pain reporting is an important factor in pain management, stating “When the patient reports the pain before it is too bad, it is easier to manage.” The nurses believed that “patients verbal communication may not always be consistent with the actual intensity of pain.” Nurse Bibi stated that *“I ask them to describe their pain and rate the pain severity so I get a better understanding of what they’re feeling.”*

#### 3.1.8. Are There Any Factors or Circumstances That Make It Difficult for Nurses to Provide Adequate Pain Management?

The nurses generally felt that adequate pain management is difficult if their patient workload is beyond two patients. Additionally, if another patient’s condition is life-threatening, such as with bleeding, then the patient in pain must wait for medication. Furthermore, patients with drug addictions or drug allergies have individual needs beyond the usual treatment plan, which complicates care. Pain assessment is also difficult if there is a language barrier or the patient is unable to communicate or is disoriented, “so, you cannot use the pain scale to determine pain severity”.

#### 3.1.9. Do the Nurses Have Concerns about the Side Effects of Opioids and the Use of Opioids after Surgery, Particularly Those with a History of Substance Use?

Five of the six nurses were concerned about the side effects of opioids after surgery. They believed that adverse effects could be life-threatening, such as impaired breathing, impaired renal function, electrolyte imbalances, GI bleeding, falling, or coma. They were also concerned about minor side effects, such as drowsiness, constipation, dizziness, nausea, and vomiting. They emphasized the importance of “educating patients about side effects, particularly of opioids”. In general, the nurses agreed that opioids are needed in the first 2 days following surgery, but after 2 days, the emphasis should be on the use of non-steroidal anti-inflammatory medications and non-pharmacologic treatments. Some nurses try not to give patients with substance abuse histories opioids and make an effort to try all other alternatives for pain management. Nurse Bibi explained that *“If the patient never had pain medicines I am okay with giving them a strong medication, but those with a drug dependency, I am concerned.*” Nurse Charlotte emphasized that *“Even with a substance abuse history, patients need their pain controlled. Short-term use is not a concern, but long-term use is a worry.”*

#### 3.1.10. What Did the Nurses Wish They Better Understood about a Patient’s Pain Experience?

The nurses wished they “knew in more detail the medical history and diagnosis” of the patients. They also wished to “learn more about different cultures about how to manage pain, so that they could try to put themselves in the patient’s shoes”. One nurse felt that “at times they were only second guessing”. Nurse Alexandra wished *“I could dig into their previous problems to know if they are pain related.”*

### 3.2. Social Norms Related to Pain and Pain Management

#### How Do the Nurses Feel about Patients in Pain Who Belong to a Cultural Group Different from or the Same as Their Own?

Each of the nurses stated that regardless of the culture they were from, they gave the “same treatment and the same approach to the patients” and “treated them without discrimination based on their culture”. Yet, several of the nurses were more comfortable with patients who shared their culture or ethnicity because they had “a greater understanding of a patient from their own culture”. However, one nurse also emphasized that “despite coming from the same culture as the patient, it does not mean that all patients from a particular culture express pain or behave in the same way or feel pain the same way.” Nurse Bibi stated that *“I’m more understanding of someone from my culture because I understand why they feel that way.”* Nurse Brandon, in contrast, stated *“I’m Haitian and just because I don’t feel the need to express my pain does not mean that another Haitian person reacts the same way. So, I would treat the pain as it is presented to me.”* The nurses recognized that the expression and reaction to pain may be different based on a patient’s cultural background and that a patient’s age may make a difference. It was emphasized that nurses should *“talk to their patients to understand their culture and their preference for pain treatment*.”

### 3.3. Behaviors Related to Pain Assessment and Management

#### 3.3.1. How Does the Intensity of a Patient’s Pain Influence the Nurses’ Response Time?

The nurses reported that they “act quickly when the patient has severe pain”, “communicate with the doctor”, and “give the patient the medicine as soon as possible”. However, the nurses “have to prioritize the care of other patients if there is a crisis situation”. Nurse Clara said, *“I won’t let them go with pain for a long time, but I have to prioritize patients.”*

#### 3.3.2. When the Nurses Assess a Patient in Moderate to Severe Pain, What Is Their First Action?

All the nurses preferred pharmacological pain management for the first post-op day, but by day 2, they preferred not to administer opioids but give “a lower level of medication”. All the nurses asked the patients about their pain management preferences. The nurses reported that when a patient is in moderate to severe pain, they “take vital signs”, “check the patient’s position”, “evaluate the patient visually”, “ask about the cause of the pain”, “evaluate pain intensity using the pain scale”, and “check to see what is ordered to relieve pain”, as they do not want the patient to “suffer”. The nurses reported that depending on the severity of the pain, commonly used drugs are Toradol, Tramadol, Dilaudid, Morphine, and NSAIDS.

#### 3.3.3. How Involved Do the Nurses Think Patients Should Be in Managing Their Pain?

The nurses felt that patients should actively participate in their care. They emphasized that patients “should know how to recognize pain” as well as “what has worked for them in the past” and “tell nurses their preferences for pain management”. The nurses believed that patients need to be educated about “when they can expect pain relief after receiving pain medication”, as well as other “non-pharmacologic treatments, such as a warm shower, repositioning or lowering lights to reduce pain”. Nurse Brandon said that *“Patients should participate 100%. They should be taught how to manage the pain when they go home and, of course; whenever we give the pain medication, we tell them exactly what we’ve given them and how long it may start working.”*

#### 3.3.4. Do the Nurses Encourage the Use of Non-Pharmacological and Complementary Treatments in Pain Management Alone or in Conjunction with Medication?

The nurses preferred to use non-pharmacological methods, such as heat or ice and repositioning, with pharmacologic methods, as “non-pharmacologic methods have no side effects and risk of addiction”. The nurses mentioned the value of a calm environment with decreased noise and light. Although one nurse mentioned the use of distraction, there was no discussion on the use of music, prayer, meditation, or other complementary therapies. Nurse Clara shared that *“Sometimes, I may believe that certain treatments and complementary therapies are not effective. However, if patients believe that the treatment helps them, they should be allowed.”*

#### 3.3.5. What Are the Nurses’ Challenges and Recommendations Regarding Pain Assessment and Management?

The nurses expressed several challenges, including “lack of orders for pain medications”, “physicians not seeing patients themselves after surgery”, “not being able to reach a doctor in a short time particularly on the night shift”, “lack of coordination between nurse practitioners who cover surgical practices”, and “the workload of the nurse which limits response time to patients”. The nurses also expressed challenges when they felt the “patient is being undertreated”. Sometimes, “patients’ medications may be insufficient”, while “some providers may not accept the nurses’ assessment and expressed concerns as they advocate for the patient”. The nurses indicated the importance of “developing pain protocols so that they could administer appropriate medications based on the pain assessments”. Nurse Amanda’s recommendation is that *“All MDs and NPs need to be better educated about pain assessment and management and work closely with nurses to ensure holistic pain management.”*

## 4. Discussion

All the nurses in this study agreed that pain is a subjective experience, and a patient’s report of pain should be believed, as purported by Özveren et al. (2018) [22]. The nurses also reported that patients’ reactions to pain and pain tolerance are different, which supports the perceptions of Wray (2014) [23]. Consistent with Yolcu, Akin, and Durna (2016), the nurses further recognized that pain restricts movement, affecting the activities of daily living, and can lead to serious post-operative complications [24]. In addition, our findings are consistent with Murray-Nobles’s (2017) [25] study, who stated that although patients may exaggerate their pain levels to receive pain medication, the nurses should not judge the level of pain reported, but there is an ethical responsibility to treat pain.

The nurses believed that establishing a personal relationship with the patient was also important and wished there was more time to get to know the patient. They realized that the way a patient reacts is also different depending on the family involvement. The nurses did express distress when patients reacted to pain by screaming or being demanding or aggressive. Yet, the nurses often felt that the patients were not understanding of their workload. The nurses wished for potentially lower nurse-to-patient ratios to decrease their own work burden and respond more quickly to patients. Patients with substance abuse histories were also of concern, as they realized that the patient may have a different level of pain tolerance and require a higher dose of medication. Yet, addiction was of serious concern, with worry about negative work outcomes if the patient was overmedicated. The nurses in the post-operative setting did wish they had a greater understanding of the disease process and patients’ pain histories to guide their assessments and treatment of pain. They understood that a patient’s level of pain was also related to the extensiveness of the surgery. However, the nurses reported that their assessments focused on visually assessing a patient, and they depended on a patient’s rating of pain on the assessment scale. The nurses did not speak about conducting a comprehensive pain assessment, which includes documentation of the location of pain, quality of pain, duration, relieving and exacerbating factors, associated factors, or the meaning of the pain as per the patient [8]. They did not report conducting a physical exam, including inspection and palpation of the pain site.

The nurses did ask the patients about their preference for pain medications. However, they expressed significant concern if a patient had a strong preference, given the possibility of addiction. The nurses realized that in the first post-operative day, most patients experience moderate-to-severe pain and need IV pain medication. However, on post-op day 2, the nurses preferred to administer weaker medications by mouth, such as NSAIDS, to prepare patients for discharge home. The nurses expressed a preference for relieving pain using non-pharmacologic methods, such as positioning, walking to relieve gas, and creating an environment with minimal stimulation. However, they had limited knowledge of other non-pharmacologic pain treatments and the use of complementary therapies, such as music or distraction. Although these complementary therapies may be used in their personal or family context, this knowledge did not transfer to informing complementary therapies they could offer to their surgical patients. Midilli et al. (2019) [26] stated that non-pharmacological treatments, such as heat, ice, and repositioning, should be used in combination with medications for pain relief, as they are independent nursing practices without side effects which can be taught to patients following surgery.

Consistent with the nurses’ concerns, Şenyüz and Kocaşlı (2017) discussed the side effects of opioids used for moderate and severe pain, specifically the effects on respiration and bowel function [27]. The nurses’ behaviors indicated that they acted quickly in administering pain medications when the patients were in severe pain and were distressed if an emerging post-operative crisis with another patient delayed their response time.

With regard to social norms, such as culture, the nurses expressed greater comfort in caring for patients of their same cultural or ethnic backgrounds, as they were more “familiar” with their expectations and behaviors. These results suggest that culture does play a role in nurses’ experiences when caring for patients of the same and different cultural backgrounds, which will be addressed more fully in another manuscript. Narayan (2010) [28] believed that nurses have a cultural perspective of pain going back to their childhood and try to provide culturally acceptable pain management.

The nurses expressed concern regarding overprescribing opioids, which could “get us into trouble” if the patient experienced negative outcomes related to opioid use. In contrast, the nurses indicated that physicians and nurse practitioners needed more education on prescribing appropriate doses of pain medication. An additional challenge expressed was the medical coverage of the unit, as there was often difficulty reaching practitioners post-operatively. The nurses were upset themselves when the pain medications ordered did not sufficiently relieve pain, and the provider did not come to see the patient but questioned the nurses’ judgement. Similar to Costello (2015) [29], who emphasized that upon post-op hospital discharge, nurses have a responsibility to educate patients about the use of opioids to prevent misuse, the nurses in the study spoke about the need to educate patients about types of pain medication, when to expect relief, and side effects, as well as recommending non-pharmacological treatments.

The nurses spoke of the importance of individualized care, recognizing differences in patients’ pain experiences and levels of pain tolerance, the extent of the surgical procedure, and differences in pain perceptions and preferences based on a patient’s cultural background. Yet, juxtaposed to this perspective, each of the nurses mentioned that they were taught to “treat all patients the same”. This potentially reflects a cognitive dissonance between patient-centered individualized care verses diversity and equity. “Treating all patients the same” may lead to a “one size fits all approach”. Access to adequate pain medications is important for all ethnic and cultural groups, but in the process of “standardizing” care, there may be a risk of jeopardizing patient-centered, individualized care. In addition, given that the nurses expressed greater comfort in caring for a patient of the same cultural background, it is unclear how this may negatively impact care offered to patients of differing cultural backgrounds from the nurse. For example, if vocalization of pain is culturally accepted, a nurse from a culture that has a stoic approach to pain may interpret “screaming” as an inappropriate pain response. Therefore, further understanding of cultural differences related to pain and pain responses needs to be discussed in educational offerings related to pain assessment and management.

In reviewing the information shared by this cohort of nurses in a post-operative observational unit, several important issues have been raised: (1) the need for nurses to conduct a comprehensive pain assessment, (2) identifying the personal attitudes and preferences of nurses related to pain and pain management in comparison to patients, (3) exploring the impact of patients’ and nurses’ cultural backgrounds on pain management, (4) offering appropriate pain management in accordance with the level of pain severity, (5) using pharmacologic treatments, including opioids, non-opioids, and adjuvant therapies, and reassessment of pain following the administration of pain medications and other treatments, (6) increasing nurses’ and patients’ knowledge regarding the use of non-pharmacologic and complementary pain therapies, and (7) lastly, in consultation with pain management experts and unit management, determining the availability of non-pharmacologic and complementary therapies available during the post-operative hospitalization period.

### Limitations

This study was approved by the institutional review boards during the first 3 months of the COVID-19 pandemic, a time when all hospitals were closed to visitors and on-site research could not be conducted. Data collection was therefore delayed until a COVID Mitigation Plan could be submitted, indicating measures to limit transmission of the virus. The time frame for data collection was also shortened, as only emergency surgeries were being performed in the first few months of the pandemic. As a further limitation, the researcher was working within a specified time frame to complete her research as stipulated by the Republic of Turkey Ministry of National Education, which funded the study. The nurses were also working under the stress of the pandemic, and the recruitment of nurses was challenging as they had time constraints related to time availability for interviews. Although the sample for this study included a cohort of six nurses of varying cultural backgrounds (Caucasian, African American, and Hispanic), no Asian nurses were working on the unit at the time of data collection, which was an identified limitation. Furthermore, the study consisted of nurse–patient dyads, with 6 nurses and 12 patients, 1 of the latter representing the same cultural background as the nurse and 1 patient being of a different cultural background from the nurse. Although a sample size of 18 participants was satisfactory for a qualitative study, the sample size of nurses was limited. Given the usual small sample sizes of 10–30 participants in qualitative studies, the results are not generalizable. However, the results provide a contextualized understanding of the human experience through intensive study of a few cases. Rather than the term generalizability often associated with quantitative studies, qualitative research speaks of transferability, which is known as case-to-case translation [30]. Let it be known that the data regarding patients will be presented in a separate manuscript, as will the data comparing the attitudes, social norms, and behaviors of the nurse–patient post-operative dyads of similar and different ethnic and cultural backgrounds.

## 5. Relevance to Clinical Practice and Conclusions

Based on the results of this study, it is suggested that nurses on the post-operative unit have a greater understanding of the medical history and prior pain history of patients. In addition, nurses should receive education regarding conducting a comprehensive pain assessment, including visualization of the pain site. In continuing education seminars, perhaps in “unit huddles”, a nursing staff could discuss the challenges to pain assessment and management and discuss “case studies” representing patients who had significant pain management issues.

Further consideration should be given to the nurses’ concerns about the use of opioids and opioid addiction. Nurses should be instructed regarding the use of the World Health Organization pain model, which indicates the importance of using non-steroidal medications, as well as adjuvant medications for mild pain (0–3 intensity), then adding opioids to this regimen in the face of moderate-to-severe pain [8]. Given the opioid crisis in America, the attitudes, social norms, and behaviors of the nurses in this study indicated heightened concerns about administration of opioids and the nurses’ preference to discontinue opioid use as soon as possible. Nurses therefore need education about the appropriate and often necessary use of opioids, particularly during the post-operative period, while educating patients about misuse. Nurses must be knowledgeable about all medications that may relieve pain to provide a balanced perspective regarding pain management, while recognizing the varying needs of patients dependent on their past pain histories, surgical experiences, substance abuse histories, and cultural influences. In addition to pain medications, education should also emphasize the use of non-pharmacologic and complementary therapies. Nurses should advocate for the availability of these treatments in the post-op unit, such as the availability of headsets so that music can be used for distraction and the creation of a calming environment during the post-op period.

Given the nurse-to-patient ratios and consideration of emergencies other than pain management, the nurses expressed distress about workloads and feeling pressured when patients were extremely demanding, which may lead to an increased risk of medication error. This suggests the need for consideration of the nurse-to-patient ratios during a 12-h shift, given the types of surgeries scheduled. Written pain protocols may also be important in treating patients during the post-operative period, particularly if the prescription providers are not immediately available to write pain orders. NP coverage of the unit may ensure the availability of providers to write pain orders.

Based on the results of this study, there remains concern that differences in the cultural backgrounds of the nurse and patient may influence the quality of care offered, specifically pain management. “Treating all patients the same” may result in stereotyping, in which the attitudes, norms, and behaviors of a person are contextualized primarily by culture. Nurses should be educated about assessing a person’s degree of assimilation in American culture and their individual perspectives regarding pain and pain management. In this study, the nurses expressed the importance of being non-judgmental, non-discriminatory, and accepting of diversity, yet juxtaposed to these ideas were the comments from the nurses to “treat everyone alike”. This negates the importance of diversity of needs, attitudes, preferences, involvement in deciding care options, and providing individualized care to meet the unique needs of patients. The implications and recommendations are for an emphasis on the importance of discussions between nurses and their patients in understanding health concerns, preparing patients for the surgical and post-operative experience, and offering guidance in a range of treatment options for pain.

What does this paper contribute to the wider global clinical community? (1) It identifies the concerns of culturally diverse nurses in providing pain management for post-operative patients; (2) it indicates nurses’ challenges and recommendations regarding pain assessment and management; and (3) it highlights the issue of culture and cultural competence as it relates to standardization of pain management of patients verses individualized, patient-centered care.

## Data Availability

Not applicable.
